# UV-spectrophotometry dataset of technical lignin in solution after aging and looped measurements

**DOI:** 10.1016/j.dib.2023.109549

**Published:** 2023-09-06

**Authors:** Jost Ruwoldt, Marita Dørdal Helgheim, Mihaela Tanase-Opedal, Kristin Syverud

**Affiliations:** RISE PFI AS, Høgskoleringen 6B, 7491 Trondheim, Norway

**Keywords:** Lignin, UV-spectrophotometry, Solution stability, Biopolymers, Kraft lignin, Soda lignin, Organosolv lignin

## Abstract

This article provides UV-spectrophotometry data of technical lignin samples in solutions, which were acquired after ambient aging for up to 110 days or looped measurements on fresh solutions. UV-spectrophotometry of lignin is a useful technique, as it can a) quantify the concentration and purity of lignin in a given sample, b) determine the abundance of phenolic hydroxyl groups, and c) yield qualitative information about chemical modification of the lignin macromolecule. In addition, the technique is rapid and easy to use. Still, solutions of lignin are known to be unstable; in particular at high pH or in presence of UV-light. The data in this article may hence serve as guide in the experimental conduct and design, as it shows the reproducibility of UV-spectrophotometry measurements of lignin. Stock solutions of technical lignin were made according to previously published procedure [1]. The solutions in dimethyl sulfoxide (DMSO) were aged in 100 mL volumetric flasks with glass stopper, taking periodic samples for measurements in a Shimadzu UV-1900 UV−vis spectrophotometer. The instrument recorded the spectrum from 500 to 200 nm at 1.0 nm intervals and medium speed, using quartz cuvettes with a pathlength of 1 cm. In addition, looped measurements were conducted on fresh solutions, where the instrument repeated the spectral range of 500 to 200 nm for in total sixteen times. The latter examined solutions of technical lignin in DMSO solvent as well as in 0.2 N NaOH in water.

Specifications Table

**Table:** 

Subject	Analytical Chemistry: Spectroscopy
Specific subject area	UV-spectrophotometry of dissolved technical lignin after aging at ambient conditions or after repeated (looped) measurements
Type of data	Chart, table
How the data were acquired	Measurements were conducted on a Shimadzu UV-1900 UV−vis spectrophotometer with lignin in solution filled into quartz cuvettes, which had a 1.0 mm pathlength.
Data format	Processed raw data
Description of data collection	The 500 to 200 nm spectral range was scanned at 1.0 nm intervals and medium speed. The lignin was dissolved in DMSO solvent or water with 0.2 N NaOH, which was then filled and diluted into quartz cuvettes. Measurements were either repeated (looped) in total 16 times to probe reproducibility or obtained of a lignin in DMSO solution after aging. Aging was performed in 100 mL volumetric flasks with stopper at ambient conditions. Each absorbance spectrum was converted to the absorptivity via division by the lignin concentration (dry and ash-free).
Data source	RISE PFI AS Høgskoleringen 6B 7491 Trondheim Norway
Data accessibility	Repository name: Zenodo Data identification number: 10.5281/zenodo.8060330 Direct URL to data: https://doi.org/10.5281/zenodo.8060330 The data is listed in the file “Data_Repository_Lignin_UV.xlsx”, which can be accessed with Microsoft Excel or other compatible software. The top segment labelled “UV-Spectrophotometry of lignin after aging” corresponds to the data obtained after aging, i.e., the absorptivity values (average of four measurements on the left and standard deviation on the right) of lignin in DMSO solution after aging with the time increment in days. The bottom segment labelled “UV-Spectrophotometry of lignin after looped measurements” is the data from looped measurements, i.e., absorptivity (average of two measurements on the left and min/max values on the right) in dependence of the measurement number, which denotes the repetition number, listing both the lignin sample and solvent employed.
Related Research Article	Ruwoldt, J., Tanase-Opedal, M. & Syverud, K. Ultraviolet Spectrophotometry of Lignin Revisited: Exploring Solvents with Low Harmfulness, Lignin Purity, Hansen Solubility Parameter, and Determination of Phenolic Hydroxyl Groups. ACS Omega **7**, (2022) 46371–46383. https://doi.org/10.1021/acsomega.2c04982

## 1 Value of the Data

• The data shows the effect of aging of lignin in DMSO solvent.
• The data indicates the reproducibility and overall trend of repeated measurements on one specific lignin solution in UV spectrophotometry.
• Researchers, lab personnel, and engineers working with analytical measurements of lignin can benefit from the data, both in the context of UV spectrophotometry and analyzing solutions of lignin in general.
• The data may be used to determine how often a single measurement can be repeated without significantly changing the outcome.
• The data can be used to judge how long lignin may stay dissolved in DMSO solvent without considerably changing its properties.

## 2 Objective

This dataset was generated as part of the preliminary work for the article in reference [1]. The objective was to determine how long a lignin solution (DMSO solvent) could be stored before analysis, without significantly affecting the measured results. In addition, the objective was to test the effect of the UV measurement on the lignin solution, as exposure to UV-light can degrade the lignin and hence alter the measured results [2]. To test the latter, both neutral solution (DMSO solvent) and alkaline conditions (0.2 N NaOH in water) were evaluated.

## 3 Data description

**Fig. 1:**
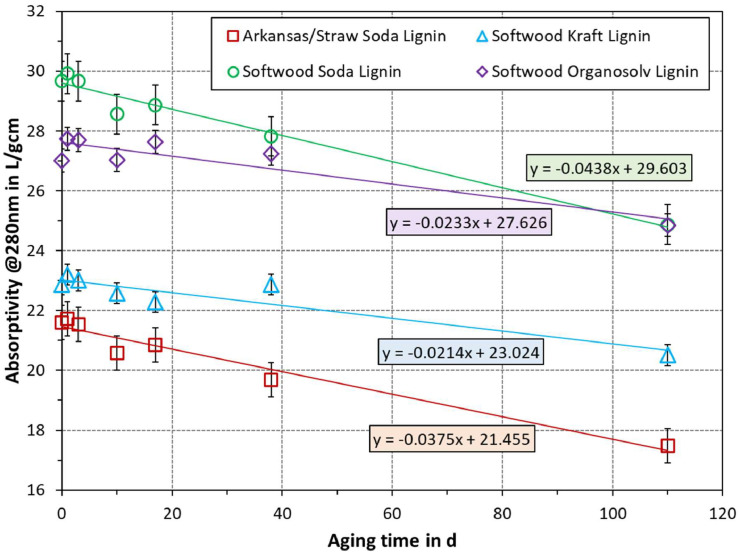
Effect of aging time of lignin in DMSO solution on absorptivity at 280 nm. Each series was fitted with a linear regression line, for which the equations are indicated in the same color. The standard deviation of each measurement is given in the error bars.

The results of aging solutions of technical lignin in DMSO solvent are plotted in Fig. 1. The linear regression lines are shown in addition, as these highlight the overall trend and provide the average decrease per day, i.e., the slope of the regression line. As can be seen, all samples showed a decrease in absorptivity over time, ranging from 0.0438 to 0.0214 L/gcm per day. The change was greatest for softwood soda lignin, followed by arkansas/straw soda lignin. Softwood Kraft lignin exhibited the smallest change, whereas softwood organosolv lignin had a marginally greater slope. The standard deviation of data points from the regression line is 0.28, 0.32, 0.28, and 0.38 L/gcm for arkansas/straw soda lignin, softwood Kraft lignin, softwood soda lignin, and softwood organosolv lignin, respectively. In the same order, the root mean square of the standard deviation (scattering) of data points for each sample is 0.25, 0.30, 0.39, and 0.21 L/gcm.

**Fig. 2:**
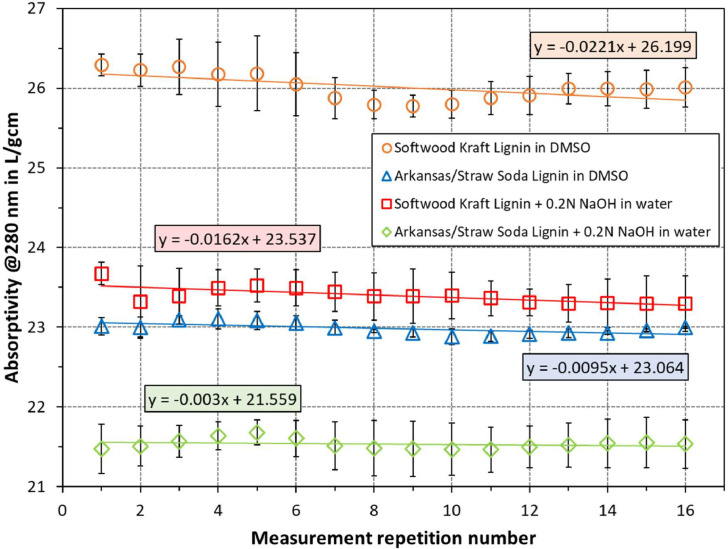
Effect of repeated (looped) measurement on absorptivity of lignin at 280 nm. Each series was fitted with a linear regression line, for which the equations are indicated in the same color. The min/max values of each measurement are given as the error bars.

The absorptivity values of repeated (looped) measurements are given in Fig. 2. Here, the overall trend was also a decrease. This change was lowest for arkansas/straw soda Lignin in water with 0.2 N NaOH, followed by the same lignin in DMSO solvent. The greatest change was observed for softwood Kraft lignin in DMSO solvent at 0.0221 L/gcm decrease per measurement on average. The individual points do not follow the linear regression line exactly; however, deviations are predominantly within the error bars. The standard deviation of data points from the regression line is 0.13, 0.06, 0.07, and 0.06 L/gcm for the softwood Kraft lignin in DMSO, arkansas/straw soda lignin in DSMO, softwood Kraft lignin in water with 0.2 N NaOH, and arkansas/straw soda lignin in water with 0.2 N NaOH, respectively. In the same order, the root mean square of the standard deviation (scattering) of data points for each data series is 0.50, 0.26, 0.52, and 0.52 L/gcm, respectively.

## 4 Experimental design, materials and methods

Chemicals were acquired as dimethyl sulfoxide (DMSO, 99.8% purity, anhydrous) and sodium hydroxide (reagent grade, ≥98%, anhydrous) from Sigma-Aldrich, Norway. Distilled water was purified via a Sartorius AriumⓇ mini water purification system before use (resistivity: 18.2 MΩ). Technical lignin samples were acquired as BioPiva 395 (softwood Kraft lignin) from UPS Biochemicals, Germany, and Protobind 1000 (arkansas/straw soda lignin) from PLT Innovations, Switzerland. Softwood soda and organosolv lignin were produced by pulping of Norwegian spruce, as stated in the previously published procedure [1]. The softwood soda lignin was precipitated with 1 M sulfuric acid from the black liquor, which was obtained after cooking the wood chips alkaline solution (liquid/wood ratio of 7.5:1 and NaOH/wood ratio of 3:10) at up to 180°C. The softwood organosolv lignin was precipitated with water from the cooking liquor (acetone/water at 50/50 volumetric ratio), which had been produced in a batch autoclave at up to 195°C (7.5:1 liquid/wood ratio).

A Shimadzu UV-1900 UV/vis spectrophotometer was used to record the UV-spectra within the range of 500 to 250 nm. The instrument used 1.0 nm increments and medium speed. Quartz cuvettes with 1.0 cm pathlength and PTFE lid were used. The reference cell was always occupied with a cuvette of the same solvent without additives. The background spectra were recorded and checked with blank solvent. Measurements on lignin solutions were only recorded, if the baseline-deviation was less than 0.005 cm^−1^. For each measurement, the stock solutions were diluted within the cuvette to achieve an absorbance of 0.3 – 1.0 cm^−1^ at 280 nm during recording. Stock solutions of 0.3 g/l lignin in 50 mL volumetric flasks were made, locked with stopper, and stored at ambient conditions for aging. Aging experiments used two dilutions with two measurements each, yielding four measurements per data point. Repeated (looped) measurements were conducted on fresh stock solutions, making two dilutions and sixteen repetitions per sample. The absorptivity was calculated by dividing the absorbance by the lignin concentration, using the dry ash-free sample weight.

## Ethics Statement

This work does not involve any studies with human or animal subjects.

## CRediT authorship contribution statement

**Jost Ruwoldt:** Conceptualization, Methodology, Validation, Formal analysis, Investigation, Data curation, Writing – original draft, Writing – review & editing, Visualization, Supervision, Project administration, Funding acquisition. **Marita Dørdal Helgheim:** Investigation, Data curation, Writing – review & editing. **Mihaela Tanase-Opedal:** Methodology, Resources, Writing – review & editing. **Kristin Syverud:** Supervision, Writing – review & editing.

## Data Availability

UV-spectrophotometry dataset of technical lignin in solution after aging and looped measurements (Original data) (Zenodo)
